# Complementary Medicine for the Modification of Risk Factors for Cognitive Impairment

**DOI:** 10.1155/2017/9472859

**Published:** 2017-02-19

**Authors:** Genevieve Z. Steiner, Sai Wang Seto, Yiu Wa Kwan, Crystal Haskell-Ramsay, David A. Camfield

**Affiliations:** ^1^National Institute of Complementary Medicine (NICM), Western Sydney University, Penrith, NSW 2751, Australia; ^2^School of Biomedical Sciences, Faculty of Medicine, The Chinese University of Hong Kong, Shatin, New Territories, Hong Kong; ^3^Brain, Performance and Nutrition Research Centre, Faculty of Health and Life Sciences, Northumbria University, Newcastle upon Tyne, UK; ^4^School of Psychology and Illawarra Health & Medical Research Institute (IHMRI), University of Wollongong, Wollongong, NSW 2252, Australia; ^5^Centre for Human Psychopharmacology, Swinburne University of Technology, Hawthorn, VIC 3122, Australia

There is a natural decline in cognitive function as we age, particularly in processing speed and working memory. A range of modifiable factors can increase the risk of accelerated cognitive decline including hypertension, chronic inflammation, atherosclerosis, diabetes, atrial fibrillation, stroke, and impaired central nervous system glucose regulation. Given the lack of adequate interventions for cognitive decline and dementia, it is essential that treatments with the potential to reduce the risk of cognitive impairment are thoroughly explored.

Nutraceutical and lifestyle medicines, including vitamins, herbs, supplements, physical activity, and diet, have been shown to possess anti-inflammatory, antihyperglycaemic, and antihypertensive properties, suggesting their utility in targeting the pathophysiology associated with risk for cognitive decline. The purpose of this special issue was to explore the role of such complementary medicines in the modification of risk factors for cognitive decline.

Authors contributed with a range of papers including original research and review articles spanning in vitro, in vivo, and human studies that improve the understanding of the pathology involved in cognitive impairment in older age and the development of evidence-based complementary treatment strategies for cognitive decline. This collection of works provides a snapshot (that is by no means exhaustive) of current research and some of the emerging trends in this field. This special issue features 11 papers including 4 reviews and 7 original research articles. The complementary therapies explored include nutritional supplements (2 papers), herbal and traditional medicines (6 papers), physical activity (1 paper), and electroacupuncture (2 papers). A brief description of these 11 works is detailed below.

- Clinical trials involving nutraceutical and herbal medicine interventions for people with mild cognitive impairment and dementia are reviewed in G. Z. Steiner et al. The manuscript titled “A Systematic Review of Intervention Studies Examining Nutritional and Herbal Therapies for Mild Cognitive Impairment and Dementia Using Neuroimaging Methods: Study Characteristics and Intervention Efficacy” focuses on papers that feature neuroimaging outcome measures.
- D. Chang et al. provide a detailed review of the literature on herbal medicine for vascular dementia in their paper titled “Herbal Medicine for the Treatment of Vascular Dementia: An Overview of Scientific Evidence.”
- Vascular disease, such as cerebrovascular disease and atherosclerosis are risk factors for cognitive impairment. X. Zhou et al. review the evidence on this link and promising Traditional Chinese Medicines in their paper titled “Vascular Contributions to Cognitive Impairment and Treatments with Traditional Chinese Medicine.”
- The fourth review featured in this special issue summarises the evidence on the efficacy of physical activity in reducing depression. D. C. Mathersul and S. Rosenbaum's paper is titled “The Roles of Exercise and Yoga in Ameliorating Depression as a Risk Factor for Cognitive Decline.”
- H. Macpherson et al. outline the findings of a clinical trial on older adults in their paper titled “The Effects of Four-Week Multivitamin Supplementation on Mood in Healthy Older Women: A Randomized Controlled Trial.”
- The first preclinical paper in this special issue explores the effectiveness of Huannao Yicong extract, a Traditional Chinese Medicine, in reducing tau hyperphosphorylation in Alzheimer's disease model rats. Y. Cao et al.'s paper is titled “Traditional Chinese Medicine Huannao Yicong Decoction Extract Decreases Tau Hyperphosphorylation in the Brain of Alzheimer's Disease Model Rats Induced by A*β*_1–42_.”
- X. Wang et al. report their findings from an electroacupuncture treatment on Alzheimer's disease model mice in their paper titled “Improvement of Electroacupuncture on APP/PS1 Transgenic Mice in Spatial Learning and Memory probably due to Expression of A*β* and LRP1 in Hippocampus.”
- The second electroacupuncture paper in this special issue explores the effects of this treatment on cerebral hypoperfusion model rats. C.-X. Zheng et al.'s paper is titled “Electroacupuncture Ameliorates Learning and Memory and Improves Synaptic Plasticity via Activation of the PKA/CREB Signaling Pathway in Cerebral Hypoperfusion.”
- In the paper titled “Preservation of Cognitive Function by Lepidium meyenii (Maca) Is Associated with Improvement of Mitochondrial Activity and Upregulation of Autophagy-Related Proteins in Middle-Aged Mouse Cortex” by S.-S. Guo et al., maca (a traditional Andean medicine) was found to improve cognition and behavior, and mitochondrial dysfunction in middle-aged mice.
- In an in vitro study by L. Liu et al., the effects of A*β*-induced neurotoxicity on SH-SY5Y cells are modulated by a the Traditional Chinese Medicine, Yi-Zhi-Fang-Dai formula. The paper is titled “Yi-Zhi-Fang-Dai Formula Protects against A*β*_1–42_ Oligomer Induced Cell Damage via Increasing Hsp70 and Grp78 Expression in SH-SY5Y Cells.”
- The final paper in this special issue by M. A. Akhtar et a. titled “Medicinal Plants of the Australian Aboriginal Dharawal People Exhibiting Anti-Inflammatory Activity” explores the medicinal effects of a range of Eucalyptus plants used in traditional Australian Aboriginal medicine by the Dharawal people.

